# Long-term Results from the Empowering a Multimodal Pathway Toward Healthy Youth Program, a Multimodal School-Based Approach, Show Marked Reductions in Suicidality, Depression, and Anxiety in 6,227 Students in Grades 6–12 (Aged 11–18)

**DOI:** 10.3389/fpsyt.2017.00081

**Published:** 2017-05-15

**Authors:** Peter H. Silverstone, Marni Bercov, Victoria Y. M. Suen, Andrea Allen, Ivor Cribben, Jodi Goodrick, Stu Henry, Catherine Pryce, Pieter Langstraat, Katherine Rittenbach, Samprita Chakraborty, Rutger C. Engles, Christopher McCabe

**Affiliations:** ^1^Department of Psychiatry, University of Alberta, Edmonton, AB, Canada; ^2^Strategic Clinical Network for Addiction and Mental Health, Alberta Health Services, Edmonton, AB, Canada; ^3^Faculty of Business, Department of Finance and Statistical Analysis, University of Alberta, Edmonton, AB, Canada; ^4^Red Deer Public Schools, Red Deer, AB, Canada; ^5^Department of Emergency Medicine and Public Health, Edmonton, AB, Canada; ^6^Trimbos-Institute, Utrecht, Netherlands

**Keywords:** youth, mental health, suicide, depression, anxiety, self-esteem, school, prevention

## Abstract

Here, we report on findings from a 15-month follow-up of a school-based program called Empowering a Multimodal Pathway Toward Healthy Youth (EMPATHY). This was primarily intended to reduce suicidal thinking in pre-teens, adolescents, and youth students aged 11–18 in middle schools (Grades 6–8) and high SCHOOLS (Grades 9–12). It also aimed to reduce depression and anxiety. The EMPATHY multimodal program consisted of repeated data collection, identification of a high-risk group, a rapid intervention for this high-risk group including offering supervised online cognitive behavioral therapy (CBT) program, a universal CBT intervention for those in Grades 6–8, a variety of interactions with trained staff (“Resiliency Coaches”), and referral to external medical and psychiatric services where appropriate. There were four time-points at which assessments were made: baseline, 3, 7, and 15 months. Here, we report cross-sectional findings over 15 months in a total of 6,227 students who were assessed at least once during the study period. Additionally, we report longitudinal findings from the 1,884 students who completed all 4 assessments. Our results found highly statistically significant decreases in suicidality rates, with the percentage of the total school population who were actively suicidal decreasing from 4.4% at baseline (*n* = 143 of 3,244) to 2.8% at 15 months (*n* = 125 of 4,496) (*p* < 0.001). There were also highly statistically significant reductions in depression and anxiety scores at each time-point. Thus, Mean Depression scores at baseline for the entire student population were 3.73 ± 3.87 (*n* = 3,244) at baseline and decreased to 3.22 ± 3.52 (*n* = 4,496) (*p* < 0.001). Since most students were not depressed, whole population changes such as this may indicate impact in many areas. In the longitudinal analysis of students who completed all four assessments, there were also highly statistically significant improvements in depression and anxiety scores at all time-points. For example, depression scores decreased from a mean of 3.43 ± 3.67 (*n* = 1,884) at baseline to 2.95 ± 3.53 (*n* = 1,884) at 15-months (*p* < 0.001), while the number who were actively suicidal decreased from 69 to 37. These results suggest that school-based multimodal programs, utilizing a combination of interventions, can have meaningful benefits across entire school populations.

## Introduction

It is widely recognized that depression is common in those aged 11–17, with up to 10% of this group meeting criteria for depression, with diagnostic rates possibly increasing (1–3). Those within this age range are referred to, depending upon their exact age, as children, “pre-teen” children, adolescents, or youth (particularly those aged at least 13). This age range (from 11 to 17) is also the time at which many psychiatric disorders first appear (4). Tragically, suicide rates are also higher in this group (5, 6), with two large studies suggesting that 4–7% of pre-teens, adolescents, and youth have made at least one suicide attempts in the previous 12 months (7, 8). Those who have the highest risk for subsequent suicide appear to be pre-teens, adolescents, and youth students aged 11–18 who have previously harmed themselves and/or who have depression (9).

Addressing this issue is therefore critical, and there are several approaches that may help based on cognitive behavioral therapy (CBT) interventions primarily, although other approaches have been found to be helpful as well (10–13). Nonetheless, the research evidence to date has not clarified if it is more effective to target entire populations (so-called “universal” interventions) (14–17), or whether it is more effective to identify and then intervene in a smaller “high-risk” group (18–21). Overall, research findings and reviews have been supportive of a variety of both high-risk and universal interventions potentially reducing suicide rates in those aged 11–18, without definitively determining if one approach has better outcomes (22–27). For this reason, some have suggested that the most effective method to address depression and suicide in pre-teens, adolescents, and youth is to combine both universal programs as well as screening for those at highest risk, followed by targeted interventions (5, 28–35). It is also accepted that schools are the most appropriate setting to screen and intervene for those aged 11–18, including to increase resiliency against both depression and suicidality (17, 36–39). Therefore, combining both universal and high-risk approaches in schools may offer the potential to offer the most positive outcomes (40, 41). Supporting such an approach, a recent review and meta-analysis concluded that future “refinement of school-based prevention programs has the potential to reduce mental health burden and advance public health outcomes” (28).

We worked with a school district in Alberta, Canada, to help them design a new program to be given during designated “health” classroom time, with the intention being to try and improve resiliency against depression and to reduce suicidal thinking in their students. This was of particular relevance to them since this school district had experienced a sharp increase in the number of youth suicides. We suggested a multimodal approach to potentially best address this issue, including regular assessments of students’ progress and thinking, including the potential for subsequent clinical interventions and referral (following informed consent). Studying complex interventions is problematic and the present study is neither randomized nor controlled, which would have been unethical when studying youth who are suicidal, but instead uses an interrupted time series design which may be appropriate for youth participation (40). This entire approach we termed Empowering a Multimodal Pathway Toward Healthy Youth (EMPATHY) (41). Previously, we have reported initial 3-month outcomes from the EMPATHY program, which found a highly significant short-term reductions in suicidality combined with improvements in both depression and anxiety across the entire school district population (41). Here, we report on the longer term outcomes in a larger group of students that were took part in this school-based EMPATHY program over a 15-month period, focusing on the key outcomes of suicidal thinking, depression, and outcome.

## Materials and Methods

### Program Location and Timing

The EMPATHY program took place in Red Deer, AB, Canada, a small city with a population of approximately 100,000 people. Sadly, in 2012 and early 2013, there had been a significant number of youth suicides in students at Red Deer Public Schools, and the community was looking for alternative approaches to try and reduce future student risks for this. We engaged with Red Deer Public Schools in May of 2013 and a widespread multi-sectoral community partnership approach was implemented involving education services, primary health care, specialist mental health care, social services, as well as others involved with youth (such as the police services). Following extensive consultation with large numbers of interested parties, researchers, and community groups, implementation of this new program (which we refer to as the EMPATHY program) started across the entire school district in February 2014, with final data being collected in June 2015 as program funding was terminated suddenly and unexpectedly by the newly elected Provincial Government. Here, we present the longer term outcomes, which consisted of data collected by the school district at four separate assessment time-points from February 2014 until June 2015. These were baseline (February/March 2014), at 3 months (May/June 2014), at 7 months (September/October 2014), and at 15 months (April/May 2015). Because of the unexpected and sudden termination determination of other, previously planned, outcomes of potential interest are not available. These included possible changes in educational attendance and educational outcomes, as well as longer term follow-up for mood and suicidality, are not available.

The program was carried out in all of the nine schools educating those aged 11–18 (Grades 6–12) located within Red Deer Public School district. Schools involved were three middle schools for those aged 11–14 (Grades 6–8); three schools that had a wider range of grades in the school buildings, but only those in Grades 6–8 at these schools were included in the study; one special school for those aged 15–18 (Grades 9–12); and two high schools for those aged 15–18 (Grades 9–12) (Table 1).

**Table 1:** **Number of students screened for depression in each grade at each time-point**.

Depression	Baseline	3 months	7 months	15 months
Grade 6	435	434	711	719
Grade 7	412	433	712	700
Grade 8	389	428	632	623
Grade 9	523	523	722	669
Grade 10	572	563	677	620
Grade 11	493	477	737	653
Grade 12	420	370	664	512

#### Assessment Tools

For depression, the EMPATHY program utilized questions from the 9-item patient health questionnaire (PHQ-9) (42), adapted for adolescents (PHQ-A), which has been well validated in youth (43–45), but which were very slightly modified from the original publications (Table 2). Suicidal risk was assessed using the questions in the PHQ-A with two separate questions: “over the past 2 weeks how often have you been bothered by thoughts of hurting yourself”; and “over the past 2 weeks how often have you been bothered by thoughts that you would be better off dead” (41) (Table 2). If a student answered positively to the first question, then two other subsequent questions were asked: “Has there been a time in the past month when you have had serious thoughts about ending your life?”; and “Have you ever, in your WHOLE LIFE, tried to kill yourself or made a suicide attempt.” These questions were asked since previous research has indicated that positive answers may indicate a higher future risk for future self-harm (9, 46).

**Table 2:** **List of questions asked**.

Source of question^a^	Question number	Stem questions (where appropriate)	Individual questions	Scoring range for each question
**List of questions asked to determine depression score and suicide risk**				
9-Item patient health questionnaire (PHQ-9)	1	Over the past 2 weeks, how often have you been bothered by	Little interest or pleasure in doing things	0–3
PHQ-9	2	Over the past 2 weeks, how often have you been bothered by	Feeling down, depressed, or hopeless	0–3
PHQ-9	3	Over the past 2 weeks, how often have you been bothered by	Trouble falling or staying asleep, or sleeping too much	0–3
PHQ-9	4	Over the past 2 weeks, how often have you been bothered by	Feeling tired or having little energy	0–3
PHQ-9	5	Over the past 2 weeks, how often have you been bothered by	Poor appetite or over eating	0–3
PHQ-9	6	Over the past 2 weeks, how often have you been bothered by	Feeling bad about yourself or that you are a failure or have let yourself or your family down	0–3
PHQ-9	7	Over the past 2 weeks, how often have you been bothered by	Trouble concentrating on things, such as reading or watching TV	0–3
PHQ-9	8	Over the past 2 weeks, how often have you been bothered by	Moving or speaking so slowly that other people could have noticed. Or the opposite-being so fidgety or restless that you have been moving around a lot more than usual	0–3
PHQ-9^b^	9	Over the past 2 weeks, how often have you been bothered by	Thoughts of hurting yourself	0–3
PHQ-9^b^	10	Over the past 2 weeks, how often have you been bothered by	***Thoughts that you would be better off dead***^c^	0–3
PHQ-9	11	If you checked off “any problems,” how difficult have these problems made it for you to do your work, take care of things at home, or get along with other people?		0–3
PHQ-9	12	Only if scored 1, 2, or 3 on question 9 does this question get asked	***Has there been a time in the past month when you have had serious thoughts about ending your life?***^c^	Yes or no
	13	Only if scored 1, 2, or 3 on question 9 was this question asked	***Have you ever, in your WHOLE LIFE, tried to kill yourself or made a suicide attempt?***^c^	Yes or no
			**Maximum possible score**	33
**List of questions asked to determine anxiety score**^d^				
HAD scale	1		I feel tense or wound up	0–3
HAD scale	2		I get a sort of frightened feeling as if something bad is about to happen	0–3
HAD scale	3		Worrying thoughts go through my mind	0–3
HAD scale	4		I can sit at ease and feel relaxed	0–3
HAD scale	5		I get a sort of frightened feeling like butterflies in the stomach	0–3
HAD scale	6		I feel restless and have to be on the move	0–3
HAD scale	7		I get sudden feelings of panic	0–3
			**Maximum possible score**	21

*^a^The original source of most of the questions used was the PHQ-9 (45)*.

*^b^While we asked these two questions separately, they are a single question in the original PHQ-9*.

*^c^The three questions in **bold and Italics** were used to determine suicide risk*.

*^d^The original source of the questions used was the 7 items regarding anxiety contained within the Hospital Anxiety and Depression Scale (47)*.

To assess the degree of anxiety, questions were included from the anxiety section of the Hospital Anxiety and Depression Scale (47) (Table 2), as these have previously been used to measure anxiety in youth in several studies (48–50).

### Multimodal Components Included in the EMPATHY Program

The multimodal program consisted of repeated data collection, identification of a high-risk group, a rapid intervention for this high-risk group including offering supervised online CBT program, a universal CBT intervention for those in Grades 6–8, non-specific interactions with Resiliency Coaches, and referral to external medical and psychiatric services where appropriate (41).

#### Data Collection

All data collection was carried out on dedicated electronic tablets within a 25-min period occurring during a standard classroom health lesson. Students logged on using only their student IDs. Electronic data collection complied with all privacy and security requirements. Questions were presented to students in a randomized order, and no data were stored on the tablets as they were directly linked to the school intranet. The data were stored in a dedicated and secure database within the Red Deer Public School system, in the same manner as all other confidential student information. Because assessments were collected electronically, it was possible for school staff to identify those potentially as “at risk” based on their scores. It should be noted that students were identifiable to school staff only by a unique study number assigned when assessments occurred, and only if the student was flagged would the school staff be able to determine the student identity. This was also the only time that information about individual student results and scores was available to school staff. Apart from these specific instances, information about individual student results and scores was not available to school staff.

All students completed questionnaires for five separate areas of interest: (1) depression (including questions on suicidal thinking); (2) anxiety; (3) use of drugs, alcohol, and tobacco; (4) self-esteem; and (5) quality of life. A measure combining all of these measurements, the so-called “EMPATHY scale,” was also captured. Measurement occurred at Baseline (prior to any other interventions), and was repeated at 3, 7, and 15 months. In the present publication, only the data on suicidal thinking (“suicidality”), depression, and anxiety are considered. Other data will be published elsewhere subsequently.

#### Identification of the “Actively Suicidal” Group and “High-Risk” Group

**Figure 1:**
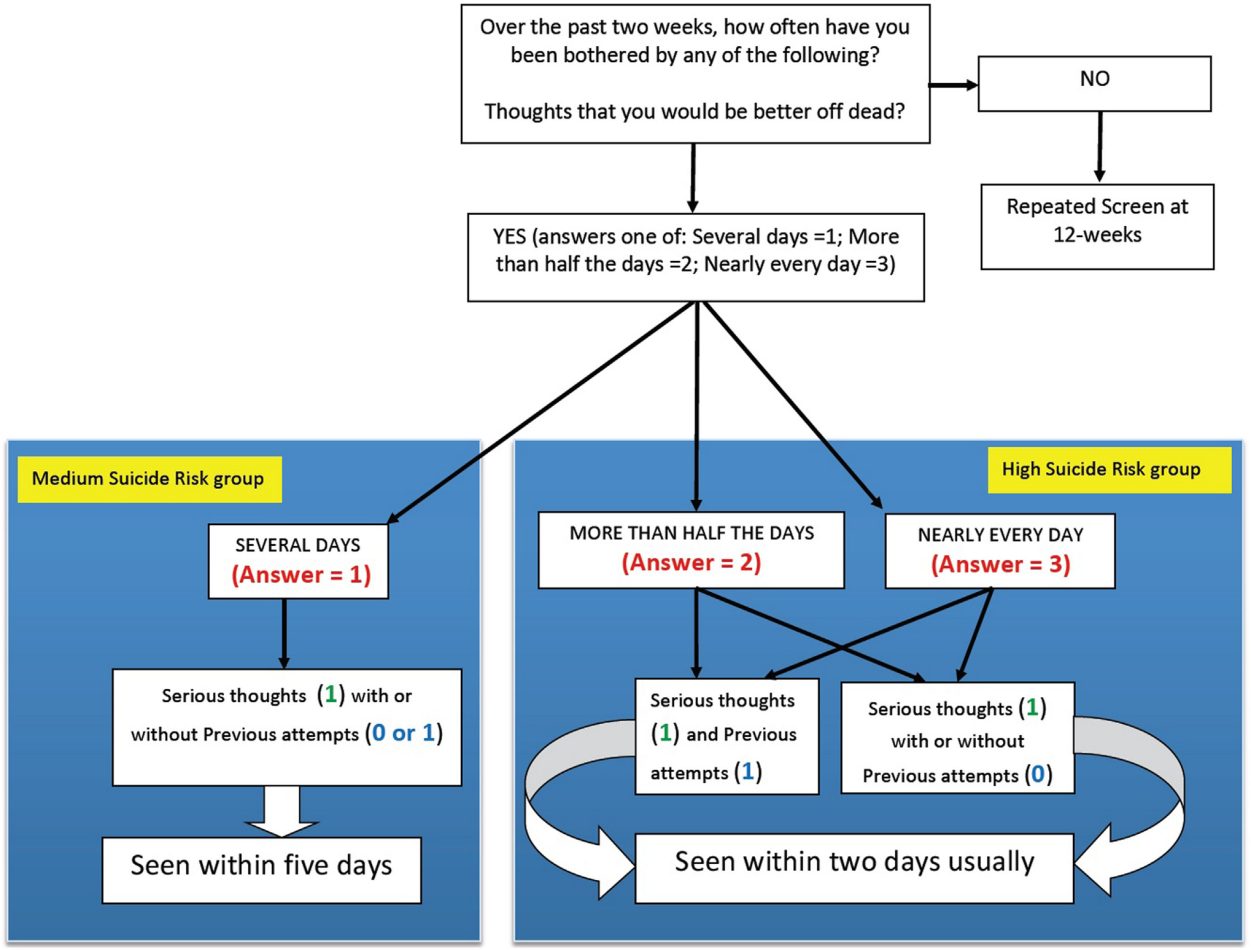
**Algorithm for determining which students were in the “actively suicidal” group**. Shows how scores on the questions regarding suicide risk determined if the student was in the high suicide risk group or the medium suicide risk group. Together, these students were considered the “actively suicidal" group, and were interviewed individually within 2 days in most cases, and everybody had been interviewed within 5 days of completing the questionnaire.

After each assessment, the results were rapidly available. From the responses to the questions on potential self-harm, students were placed in a category of suicide risk being either (i) none, (ii) low, (iii) medium, or (iv) high suicide risk (Figure 1). Those considered at “Higher risk” (i.e., those in the (iii) medium suicide risk group or (iv) high suicide risk group based on their scores) were identified to school staff. It should be noted that *a priori* it had been agreed that any student who scores indicated they were either in the high suicide risk group or the medium suicide group would be considered “actively suicidal.” Those students who were deemed “actively suicidal,” as well as the group with the highest scores on the depression and anxiety rating scales, were considered the “High Risk” group.

#### Rapid Intervention and Focused CBT Intervention

Students who were identified as belonging to the “High Risk” group were interviewed in school hours and their families were subsequently contacted, usually within 2 days of data completion (in 83% of cases). All others in this group were interviewed and families were contacted within 5 days. This interview was performed by a trained member of staff (usually somebody familiar to the student). This took the form of a 1-h semi-structured interview, during which there was a more detailed assessment of suicide risk (enhanced by previous training of school staff). There was also an open-ended discussion of issues that were relevant to the individual student. Following this interview, the student’s parents (or guardian) was contacted by school staff and informed that there were concerns. At this time, a plan was agreed upon with the parent (or guardian), which could include referral to Emergency Room, specialized Mental Health services, or their primary care physicians.

This group was also provided with information regarding the possibility of taking part in guided CBT approaches that have previously been recognized as clinically effective in this age group (51–54). However, only those students whose parents signed an informed consent form, and where the student also completed an assent form (as this was not part of the regular school program), had access to this additional program. It was also emphasized to both the student and parent that subsequent support and treatment was independent of whether or not the student took part in any subsequent CBT study.

#### Universal Intervention—OVK

The second intervention was only for students in Grades 6–8 (mean ages 11.3 at baseline in Grade 6, 12.3 in Grade 7, and 13.3 in Grade 8). This was a “Universal” intervention, since all students in these grades received this CBT-based interactive program designed to reduce rates of depression, which was given during regular classroom time. This program is an updated version of the Penn Resiliency Program (55–58) which was modified and used in the Netherlands, and it is referred to by its Dutch initials (“OVK”) (59–62). The school district translated and modified this program to better suit their students (in collaboration with the authors of OVK), and it was administered by the Resiliency Coaches after appropriate training (41). However, it should be noted that in the first year it was given as a limited version consisting of the initial 8 CBT sessions, while in the second year, all students were given a full set of 16 sessions, the initial 8 CBT sessions followed by the second 8 sessions focused on social and educational learning.

#### Resiliency Coaches

All staff hired for the EMPATHY program had experience working with youth but were deliberately chosen not to be highly qualified individuals (thus excluding psychologists, nurses, or teachers for example). This was to determine if the EMPATHY program could be successful with staff who could potentially allow the program to scale up, recognizing the potential shortage (and cost) of more highly trained individuals. These individuals were termed “Resiliency Coaches,” and each was attached to a specific school, but were not therapists or counselors, and did not act in those roles. They carried out all screening, supervised CBT programs and were available during unstructured student time. As such, the resiliency coaches were encouraged to be active and available during many periods when they could have spontaneous interactions with any student that wanted this. This occurred during breaks, play time, in addition to scheduled sessions. There was no formal measurement of the amount of interventions or interactions, but Resiliency Coaches were highly visible resources available to all students to access if they chose.

#### Referral to External Medical and Psychiatric Services

We worked closely with local services for both family practice and specialist psychiatric care. This included dedicated training on diagnosis and treatment approaches for community physicians and mental health staff working in primary care. We also provided specific training on CBT and other treatment approaches for youth. We worked to support the local mental health providers to ensure that if a large number of students were identified as needing support, appropriate resources would be available to help treat individuals identified by this program. During the study, careful tracking of all referrals to both primary care and specialist mental health care was carried out. In fact, in the first phase of the program only 60 students (2% of total screened) required external referral during the first 24 weeks (41).

#### Statistical Analysis

Since the data showed evidence of non-normality, non-parametric tests were carried out to compare the differences between the median scores at baseline and each follow-up. In all tests we used a significance level of α = 0.05. For the statistical tests on all students screened, we used Wilcoxon rank-sum tests to test the equality of medians from two independent groups. For the statistical tests on students who completed all 4 ratings, we used Wilcoxon signed-rank tests (paired). Here, we assume the sample is a paired design, in which each student who completed both baseline ratings and follow-up ratings was their own control. A statistical power analysis had been completed prior to our initial study (41), which determined that the study was adequately powered.

Statistical analysis was carried out on an “intention to treat” basis utilizing R, version 3.1.0 (R Foundation for Statistical Computing, Vienna, Austria) and Stata/IC 13.1 for Windows (StataCorp, College Station, TX, USA). Correlations were calculated using IBM SPSS Statistics 20.0 (Chicago, IL, USA).

Note that although individuals measurements of self-esteem, quality of life, and use of drugs, alcohol, and tobacco, were made, as was a summary scale, all of these issues will be considered separately in other publications.

## Results

**Figure 2:**
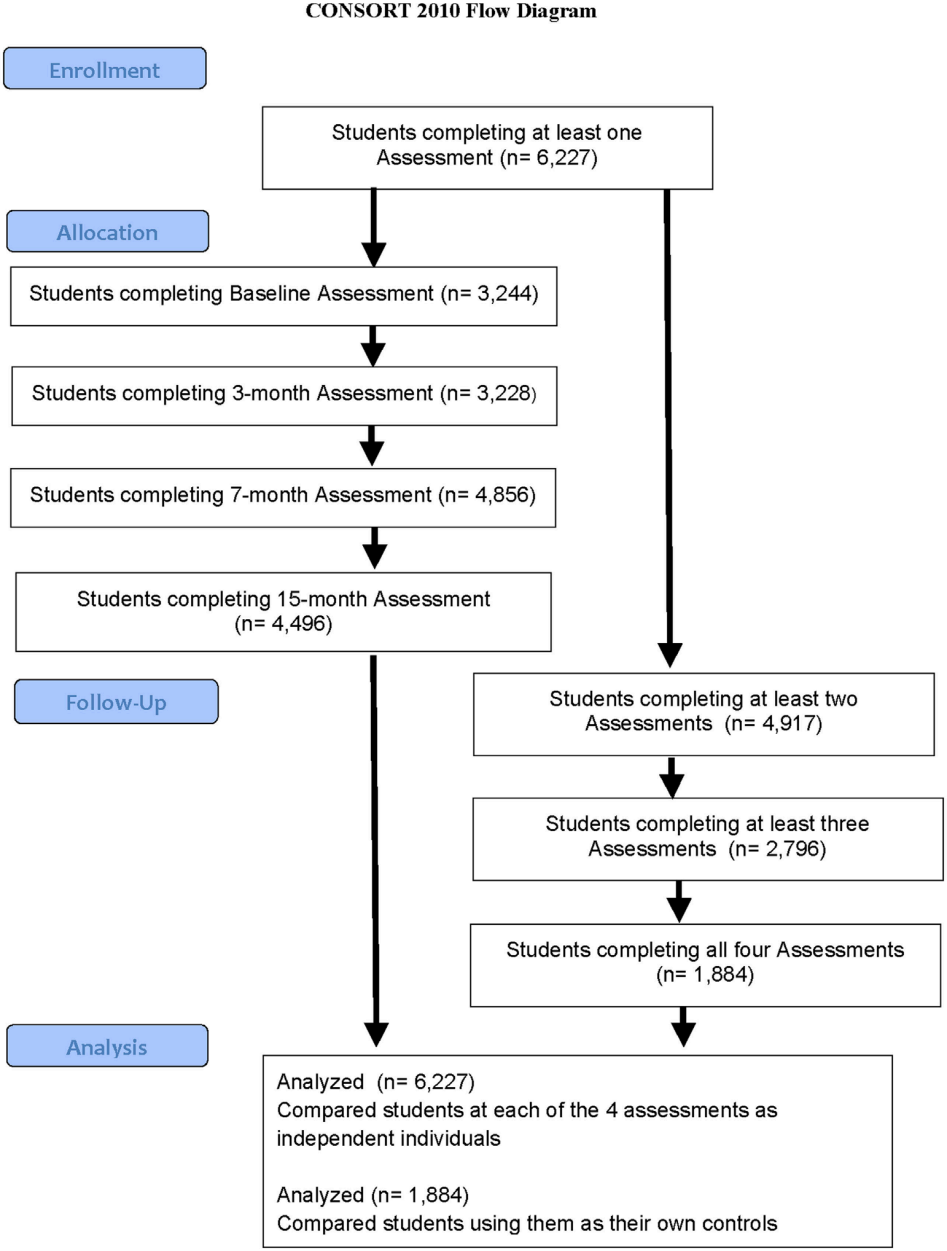
**Flowchart demonstrating assessment completion by students**. Shows how many students completed an assessment at baseline, 3, 7, and 15 months (on the left-hand side) as well as how many students completed multiple assessments (on the right-hand side).

Assessment #1 (Baseline) occurred during February and March 2014 (*n* = 3,244); Assessment #2 was the 3-month follow-up screening which occurred during May and June 2014 (*n* = 3,229); Assessment #3 was the 7-month follow-up screening which occurred during September and October 2014 (*n* = 4,860); and Assessment #4, the final 15-month follow-up assessment, occurred during the period April to June 2015 (*n* = 4,496). The timing of the follow-up screenings were based around the school year, with Assessments #1 and #2 in school year 1, and Assessments #3 and #4 occurring in school year 2, with all schools being on vacation in July and August 2014. Note that only five schools took part in school year 1, the three middle schools and two high schools. Of these students, a total of 6,227 fully completed at least one assessment, 4,917 completed at least two assessments, 2,796 completed at least three assessments, while 1,884 completed all four ratings (Table 1; Figure 2). There were an additional 324 students who signed in but then did not complete any assessments and were not therefore included in the analysis. Thus, a total of 6,651 students potentially were involved, but we have data on only 6,227. Of the total group of 6,651 students, 2,121 identified as males (32%), 2,273 identified as females (34%), while 2,257 students (34%) declined to identify their gender. For this reason, we did not analyze compare male and female data.

Of the 1,884 students who completed all four assessments over both years, this group consisted only of students in Grades 6–11 from the first school year. This is because they could not be in either Grade 6 in school year 2 (as they would be new to the system) or Grade 12 in school year 1 (as they would have graduated before school year 2). The number of students who completed Depression ratings for each grade at each time-point is shown in Table 1, and it can be seen that there were more participants in every grade in the second year as more schools were involved.

### Depression and Anxiety Scores in Total Study Population

**Figure 3:**
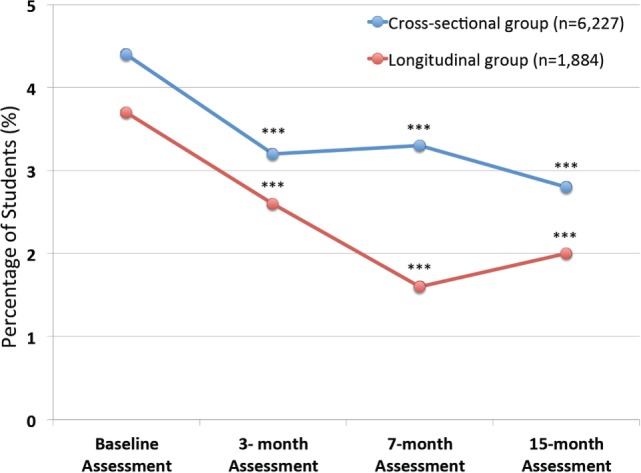
**Percentage of students deemed “Actively Suicidal” at each assessment**. Shows the percentage of students who were deemed “actively suicidal” according to defined criteria at each assessment. Data are shown both for cross-sectional group of 6,227 who completed at least one of the 4 assessments (Baseline *n* = 3,244; 3-month assessment *n* = 3,228; 7-month assessment *n* = 4,856; and 15-month assessment *n* = 4,496), as well as for the 1,884 students who completed all 4 assessments and consist of the longitudinal group. It can be seen that there are highly significant differences from baseline assessments in both groups at all subsequent assessments (***p < 0.001).

At baseline, the scores for the total of 3,244 students who completed the depression and anxiety scales varied by age (or Grade) as we have previously shown (41). However, in the present study, we found a highly significant decrease in suicidal thinking, both in terms of the percentage who were actively suicidal (either high suicide risk or medium suicide risk) (Figure 3) as well as those who had a higher suicide risk (Table 3). This may suggest that the multimodal EMPATHY decreased the risk of suicide, although data regarding actual suicide rates in the region are not available at this time-point. Additionally, scores for all students for both depression and anxiety significantly decrease over time, even though the vast majority were not depressed or anxious at baseline (Table 4). This may suggest that the EMPATHY program had a beneficial effect on the entire school population.

**Table 3:** **Changes in suicidality for entire study population**.

Level of suicide risk^a^	Baseline Assessment #1 (*n* = 3,244)	3-month Assessment #2 (*n* = 3,228)	7-month Assessment #3 (*n* = 4,856)	15-month Assessment #4 (*n* = 4,496)
High suicide risk (*n*)	81	64	76	61
Medium suicide risk (*n*)	62	40	85	64
**Actively suicidal (%)**	**143 (4.4%)**	104 (3.2%)***	161 (3.3%)***	125 (2.8%)***
Low suicide risk (*n*)	91	86	125	101
**Any suicide risk (%)**	**234 (7.3%)**	190 (5.9%)***	286 (5.9%)***	226 (5.0%)***

*Findings in bold indicate key outcome measures*.

*^a^According to algorithm (41)*.

***p < 0.001 compared to baseline.

**Table 4:** **Changes in Depression and Anxiety Scores for entire study population**.

	Baseline Assessment #1 (*n* = 3,244)	3-month Assessment #2 (*n* = 3,228)	7-month Assessment #3 (*n* = 4,856)	15-month Assessment #4 (*n* = 4,496)
Mean Depression score (±SD)	3.73 (±3.87)	3.29 (±3.61)***	3.34 (±3.54)***	3.22 (±3.52)***
Mean Anxiety score (±SD)	6.95 (±4.70)	6.32 (±4.89)***	6.44 (±4.85)***	6.21 (±4.86)***

***p < 0.001 compared to baseline.

### Suicidality, Depression, and Anxiety Scores in Those Who Completed All Four Ratings

A total of 1,884 students completed all 4 assessments and therefore constitute a longitudinal follow-up group (Tables 5 and 6). As with the total study population, the scores for depression and anxiety significantly decreased at each time-point compared to baseline (Figure 3), as well as having highly significant decreases in the percentage of those who were actively suicidal. Thus, there was a decrease of nearly 50% in the number of individuals who were actively suicidal compared to baseline, from 69 to 37 (Table 5).

**Table 5:** **Changes in suicidality for study population of 1,884 who completed all 4 ratings**.

Level of suicide risk^a^	Baseline Assessment #1 (*n* = 1,884)	3-month Assessment #2 (*n* = 1,884)	7-month Assessment #3 (*n* = 1,884)	15-month Assessment #4 (*n* = 1,884)
High suicide risk (*n*)	39	30	11	16
Medium suicide risk (*n*)	30	19	19	21
**Actively suicidal (%)**	**69 (3.7%)**	49 (2.6%)***	30 (1.6%)***	37 (2.0%)***
Low suicide risk (*n*)	48	35	27	36
**Any suicide risk (%)**	**117 (6.2%)**	84 (4.5%)***	57 (3.0%)***	73 (3.9%)***

*Findings in bold indicate key outcome measures*.

*^a^According to algorithm (41)*.

***p < 0.001 compared to baseline.

**Table 6:** **Changes in Depression and Anxiety Scores for study population of 1,884 who completed all 4 ratings**.

	Baseline Assessment #1 (*n* = 1,884)	3-month Assessment #2 (*n* = 1,884)	7-month Assessment #3 (*n* = 1,884)	15-month Assessment #4 (*n* = 1,884)
Mean Depression score (±SD)	3.43 (±3.67)	2.95 (±3.37)***	2.83 (±3.25)***	2.95 (±3.29)***
Mean Anxiety score (±SD)	6.72 (±4.64)	5.95 (±4.77)***	5.78 (±4.77)***	5.98 (±4.87)***

***p < 0.001 compared to baseline.

## Discussion

The present results demonstrate that a complex multimodal intervention can impact entire school populations in a positive manner. The results build on the initial 3-month findings we have previously reported (41) and demonstrate that there can be significant longer term improvements in the rates of suicidality, depression, and anxiety across large school populations. Given that the majority of students did not have depression or anxiety, the ability to demonstrate such changes on a school-wide basis suggests that these are meaningful. Supporting this suggestion was the finding that the percentage of students who were actively suicidal decreased from 4.4% of the entire school population at baseline to 2.8% when the EMPATHY program ended. This positive improvement occurred despite the fact that most students received no direct interventions, i.e., those in Grades 8–12 did not receive resiliency training, and most students in all Grades did not have guided Internet CBT interventions.

It is possible that several components that may have contributed to these findings. First, since students received rapid feedback after competing questionnaires, with interviews in most cases being less than 48 h in most case, this may have been impactful. Anecdotal feedback from several students was that this was seen as very positive, as the rapid feedback made them feel that their issues were taken seriously, an idea which has previously been reported (63). Again, anecdotal feedback suggested that friends of the individuals identified also gained positively as they now were aware of additional support. Additionally, involvement of parents may also have been a contributing factor (64, 65). In this instance, there was extensive anecdotal feedback from parents who were extremely positive about how this process helped their understanding of the issues their pre-teen, child, or youth was experiencing. The impact of other, somewhat similar, programs on school cultures has been reported previously (66–68). However, determining if this occurred would have required specific qualitative study which was not carried out.

An additional possibility is that improvements were due, in part, to Resiliency Coaches spending a lot of recreational time in the same environment as the students and, while they were specifically trained to not act as “therapists,” they found that on many occasions students would spontaneously approach them and sometimes confide in them. This included disclosures regarding physical and/or sexual abuse they had experienced or witnessed. The impact of this is uncertain, but this is a different role to that held by specific school counselors, and the Resiliency Coaches were surprised by how frequently their availability allowed such spontaneous interactions to occur. It is recognized that friendship skills can help adolescent depressive symptoms in combination with other approaches (69), so it is possible this may also have played a role. Currently, however, these possibilities all remain speculative. In future studies, it will be important to examine them more specifically to determine what, if any, impact each of these may have.

In terms of the potential benefit for a universal CBT intervention (OVK), this remains uncertain. Given that in the present study, it was used in a different and truncated manner in the first year and administered by a different group of individuals than those who have previously been shown to deliver it effectively, it cannot be considered to have been adequately tested in the present study. That universal interventions may be more effective when delivered by psychologists has been suggested from other studies (70) means that we are uncertain what our negative findings may indicate. Determining the relative importance of this aspect of the program will be required in future studies, and most importantly, it is necessary to study the effectiveness of the full 16-session program. Nonetheless, it is well recognized that it can be difficult to detect the overall effect of such universal interventions, and this may explain the large number of studies that are unable to show a benefit. The potential benefit of universal approaches such as OVK, as well as those recently examined for suicide prevention (27, 33), needs to be evaluated as part of the approach of combination programs such as EMPATHY, but are unlikely to be measures that on their own significantly reduce youth depression and suicide rates.

In terms of the measurement scales used, it is important to recognize that simply using depression rating scales on their own may not be sufficient. This is because many pre-teens, children, and youth aged 11–18 have “sub-threshold” depression (i.e., depressive symptoms that do not meet diagnostic criteria) (71, 72), and many of these also have active suicidal thoughts (8). For these reasons, and the fact that scales for depression do not examine previous self-harm behaviors, most standard depression screening tools likely miss many youth who have active suicidal thoughts (73). Nonetheless, it does appear that the questions contained within the PHQ-9 may be very useful as part of a screening tool (74).

A further advantage of the present findings was that we could examine findings from two different groups: first those who completed assessments at least once (*n* = 6,227), giving cross-sectional data, and second the smaller group of 1,884 students who completed all 4 assessments and therefore provided longitudinal data. Utilizing findings from both groups increases our ability to have confidence in the findings since they both had similar outcomes.

An additional, anecdotal report was that many of the teachers and school Principals reported a marked decrease in the number of bullying cases brought to their attention. Although the schools did not formally collect specific data to measure this suggestion, these findings are compatible with our previous suggestions that the positive changes found initially at 3 months (41) (and in the present study) may have been due to a combination of several interventions, as well as non-specific interactions. Further research is needed to validate any such suggestions, and if so to more accurately determine whether any specific interventions, on their own, can have similar impacts across a school district.

When comparing the present findings to those from previous research it is important to note that we included all students within a complete school district. There was no exclusion of particular schools or groups of students, both of can conceivably bias any findings. This is also different in that here we have analyzed data collected as part of a multimodal program introduced on a district-wide basis to all schools, rather than a research study in which single, or combined, factors are compared. However, such an approach leads to the significant limitation that the lack of any control groups limits conclusions that may otherwise be made. Nonetheless, since one of the challenges facing those who wish to carry out research to improve the mental state of youth is the fact that students move between schools, only by examining depression rates or suicidal thinking across entire student populations can more accurate conclusions be made.

Another potential limitation is that the EMPATHY program took place during two different school years, and it was therefore conceivable that there might have been timing effects that impacted the outcomes. Thus, there could have been a major change only in school year 1 and not school year 2, in which case there should be no improvement in scores between Assessments #2 and Assessments #3. Or, alternatively, students may improved at Assessment #2 simply because of they were close to the summer vacation but then got worse when they returned (Assessment #3), only to improve again and have good results at the end of the second school year (Assessment #4). However, the results showed no such impacts, and in particular when we examined changes between the end of the first school year and the start of the second school year (Assessments #2 and #3, respectively), we found ongoing statistically significant improvements between these two visits for depression scores. This suggests that the EMPATHY program had long-lasting benefits which continued over time, and further might imply that the more interactions the students had, the greater the benefits. This was also suggested by the findings from the longitudinal group of 1,884 students. Both of these findings suggest no strong seasonal component underlay the current findings.

Other study limitations are that children, pre-teens, and youth all develop cognitively over time. However, we had no measurements either at baseline or at 15 months regarding how they may have possible changed during the 15-month follow-up period. It is conceivable that this may have, in some non-specified manner, altered the findings. We think this unlikely, given the consistency across grades, but we cannot be certain this had no impact. For example, all the 1,884 children and youth who were followed-up repeatedly were 15 months older than at baseline age; therefore, their ability to respond to questions and ease of handling computerized assessments most probably had improved. Similarly, we have no information regarding the general cognitive ability of study participants. In any classroom cohort, there are be adolescents with learning problems, or intellectual difficulties, but we have no information about individual students and whether this impacted outcomes, and no information about gender and age dispersion in classrooms or across grades, or cognitive ability at baseline regarding readability or understanding of questions.

Our inability to discuss the data in terms of possible gender differences is also a limitation. The large number of individuals who declined to identify their gender may have been, in part, a response to the fact that there were only two choices. Clearly, future youth surveys should give greater choice to individuals as to how they choose to identify their gender, and this may increase compliance rates.

In conclusion, the need for longer term, effective programs that can reduce youth suicidality and depression remains clear (5), and although a recent review showed that many programs do have some positive benefits (28), we believe the results from the present EMPATHY study program suggest that multimodal approaches may lead to significant mental health benefits over a longer time period. The present results from this program give support to the utility of such multimodal interventions, including combining both universal and targeted approaches, although controlled studies are needed before widespread implementation can be recommended. Initially, we had planned further studies, in part to determine reproducibility both in similar and different school populations. Unfortunately, although this was initially supported, with the unexpected ending of funding it was not possible to do this. However, we strongly encourage others to do this as implementation of any major programs in youth should only be instituted after rigorous research demonstrating reproducibility.

## Ethics Statement

This program was approved by the Health Research Ethics Committee of the University of Alberta on December 5, 2013, ethics protocol number Pro00041063. Amendments to the original protocol and consent letters were approved in January of 2014, with all changes to the informed consent letter subsequently approved prior to the start of the program. The first student was screened in February 2014 and the follow-up screening was completed by June 2015. No additional student follow-up is possible, since program funding was terminated unexpectedly in June 2015 following a change in Provincial Government in Alberta in May 2015. This study is registered with http://ClinicalTrials.gov identifier: NCT02169960. Although an application for this was completed at the time of the ethics approval, due to an unfortunate administrative oversight, the actual submission to the registration database did not occur until July 2014. This omission was corrected as soon as it was recognized and was noted in our original publication (41). It should be noted upon consultation with the school district (and with the agreement of the Health Ethics Board of the University of Alberta, the supervisory ethics board), the screening process was deemed not part of the study since the school district was adamant that programs that they implement school district-wide did not need specific parental consent. This was based on the fact that education for any subject matter within these schools, even areas that may be considered controversial (such as discussions about sexual matters or sexual orientation, or ethical matters such as physician-assisted suicide) do not require parental consent. Thus, they did not believe that teaching students on a universal, district-wide basis about mental health issues required a different process than any other subject. Similarly, they asserted that assessing students on a range of topics is done frequently, again, on a universal, district-wide basis, and the assessments in the screening were not something for which specific consent was required. Indeed, they felt that requesting specific consent for what was taught or collected as part of class would be antithetical to their processes. Per their normal processes, they sent an information letter to all parents about the new program, thereby informing the parents/guardians that the school district was introducing a school district-wide “Resiliency Project” (they did not want to use the term “EMPATHY program” as they had recently introduced a different program that had a similar name). As noted, this approach was specifically agreed by the University of Alberta Health Ethics Review Board. The University of Alberta, as well as the Strategic Clinical Network for Addiction and Mental Health of Alberta Health Services, helped design the most appropriate screening tool and intervention program, train teachers about assessment, provide close liaison to clinical services in the region when required, and evaluate the effectiveness of the program. In discussions, the school set a threshold to trigger invitation to the targeted intervention, and this threshold varied by age and by school. This component included the option of taking part in a guided Internet-based CBT program. Since participation in this additional CBT program was not part of regular teaching, prior to receiving any such additional Internet-based CBT, the parents or guardians of the students provided written consent on behalf of the youth. Additionally, the student provided written assent, and all documents and the process were approved by the University of Alberta Health Ethics Review Board. The signed consent and assent forms were kept as part of the trial documentation, in a secure, private location. The data collected by the school as part of their regular classroom assessment were made available for analysis by the research team, but only on an anonymized basis. From a scientific viewpoint, the most rigorous findings would have come from a study in which the design included a group of students who were randomized to one group or another, one of which would have been to not have any intervention. Indeed, such an approach has been carried out in studies looking at depression rates in students and in theory randomization can be carried at the class level or school level. However, this was a school district-wide program that was specifically focused on decreasing the number of youth who were actively suicidal, with assessments of students to measure this. To have considered a program (or study) in which a group of students was assessed, found to be suicidal, but then not offered an intervention would clearly have been unethical. For this reason, we determined that the most appropriate methods to analyze the data collected by the school district was by examining data from both a cross-sectional analysis of all students who completed at least one assessment, as well as using a longitudinal analysis for those students who completed all four assessments (where we would be using each student as their own control). This approach to analysis of the data was approved by the Health Research Ethics Committee, with subsequent modification in April 2016 to approve secondary analysis of the information obtained from the EMPATHY program (presented here in the present publication) with the ethics protocol number Pro00061164.

## Author Contributions

PS, MB, VS, AA, JG, SH, CP, PL, KR, and RE were responsible for the design of the program. IC, SC, and CM were responsible for all statistical analysis, assisted by VS and KR.

## Conflict of Interest Statement

The authors declare that the research was conducted in the absence of any commercial or financial relationships that could be construed as a potential conflict of interest.
